# Supplementary material for: Causal Relationship between Aging and Anorexia Nervosa: A White-Matter-Microstructure-Mediated Mendelian Randomization Analysis
Source: Biomedicines. 2024 Aug 16;12(8):1874. doi: 10.3390/biomedicines12081874 (PMC11351342; doi:10.3390/biomedicines12081874)
Supplement: Supplementary file 1 [file biomedicines-12-01874-s001.zip › Supplementary Figure/Figure S4/Singe SNP plot of MR analysis for TL to ubm-b-1739.pdf]

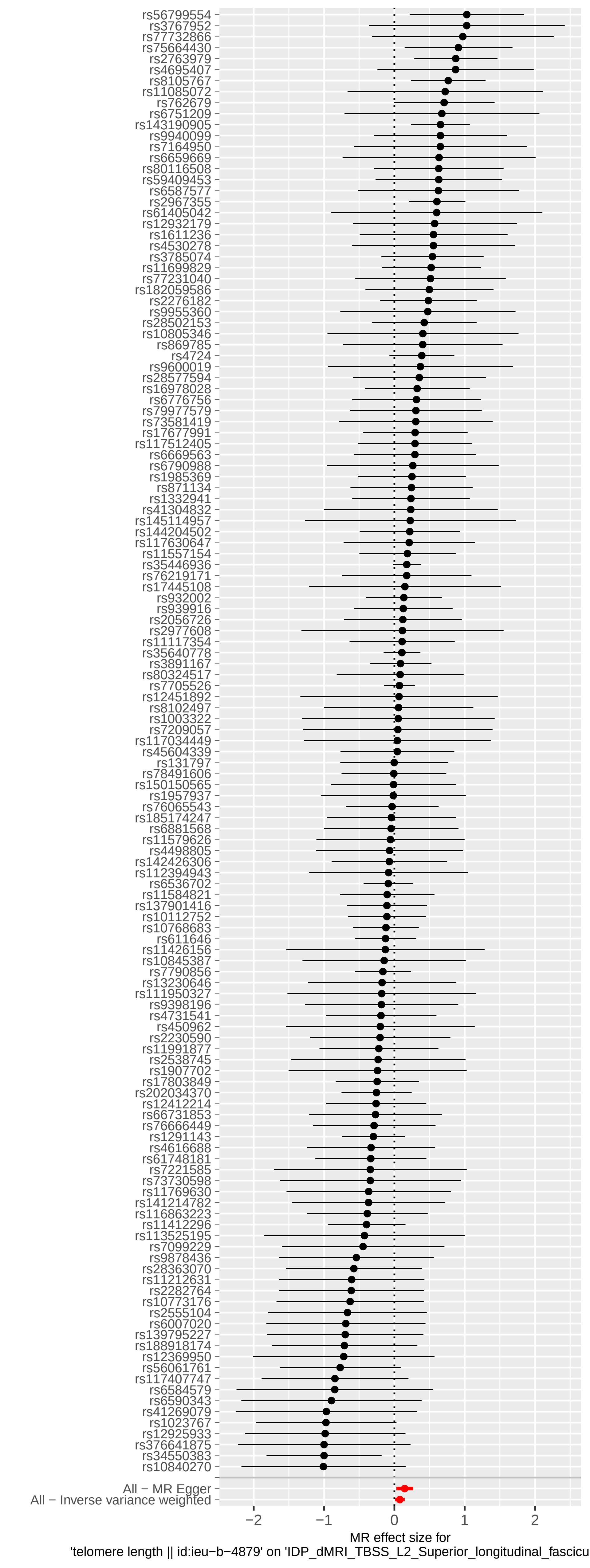
rs56799554
rs3767952
rs77732866
rs75664430
rs2763979
rs4695407
rs8105767
rs11085072
rs762679
rs6751209
rs143190905
rs9940099
rs7164950
rs6659669
rs80116508
rs59409453
rs6587577
rs2967355
rs61405042
rs12932179
rs1611236
rs4530278
rs3785074
rs11699829
rs77231040
rs182059586
rs2276182
rs9955360
rs28502153
rs10805346
rs869785
rs4724
rs9600019
rs28577594
rs16978028
rs6776756
rs79977579
rs73581419
rs17677991
rs117512405
rs6669563
rs6790988
rs1985369
rs871134
rs1332941
rs41304832
rs145114957
rs144204502
rs117630647
rs11557154
rs35446936
rs76219171
rs17445108
rs932002
rs939916
rs2056726
rs2977608
rs11117354
rs35640778
rs3891167
rs80324517
rs7705526
rs12451892
rs8102497
rs1003322
rs7209057
rs117034449
rs45604339
rs131797
rs78491606
rs150150565
rs1957937
rs76065543
rs185174247
rs6881568
rs11579626
rs4498805
rs142426306
rs112394943
rs6536702
rs11584821
rs137901416
rs10112752
rs10768683
rs611646
rs11426156
rs10845387
rs7790856
rs13230646
rs111950327
rs9398196
rs4731541
rs450962
rs2230590
rs11991877
rs2538745
rs1907702
rs17803849
rs202034370
rs12412214
rs66731853
rs76666449
rs1291143
rs4616688
rs61748181
rs7221585
rs73730598
rs11769630
rs141214782
rs116863223
rs11412296
rs113525195
rs7099229
rs9878436
rs28363070
rs11212631
rs2282764
rs10773176
rs2555104
rs6007020
rs139795227
rs188918174
rs12369950
rs56061761
rs117407747
rs6584579
rs6590343
rs41269079
rs1023767
rs12925933
rs376641875
rs34550383
rs10840270
All − MR Egger
All − Inverse variance weighted
−2
−1
0
1
2
MR effect size for
'telomere length || id:ieu−b−4879' on 'IDP_dMRI_TBSS_L2_Superior_longitudinal_fascicu
